# Medical Opinion and Movement

**Published:** 1907-06-22

**Authors:** 


### Medical Opinion and Movement.

With all our knowledge of the causes of disease and of the spread of epidemics, and our powers of preventive medicine, the Indian plague still baffles and defies us. The appalling condition of things was brought prominently before us by Mr. Morley's speech on the Indian Budget. No less than five and a quarter millions of people have perished from the disease since its first outbreak in India in 1895. Last year there were signs which gave some hope that the disease was abating, but the mortality figures for the first four months of this year are greater than for the corresponding period of any preceding year. There seems no doubt that the disease is responsible in a large measure for the disaffection among the natives. The extraordinary disparity in the numbers of natives and Europeans affected naturally arouses suspicion in their minds. Mr. Morley assures us that everything is being done that is possible to stay the ravages of the disease. One hoped that the discovery of the dissemination of the disease by rats might lead to fruitful results by their wholesale destruction. But one has always to reckon with the insanitary conditions in which the natives live, and it is with these that it is so difficult to deal and almost impossible to reform.

Dr. Busquet of Bordeaux has made an interesting investigation into the infectivity of tram-car-tickets. Taking with him some sterilised envelopes, he made several journeys on the tramways of the city, and on receiving the tickets from the conductors he tore off the corner by which he himself held the ticket and placed it in one of the envelopes. In this way he collected ten tickets from five conductors. Cultures from these tickets showed the presence of a variety of germs, such as streptococci, staphylococci, pneumococci, and the pseudo-bacillus of Loeffler. White mice were inoculated with the culture bouillon, and out of ten so treated eight died. Five white rats were also inoculated, and all died. We refer to this investigation because a member of the House of Commons considered it sufficiently important to base a question upon it to the Home Secretary, and to ask him whether he was prepared to have an analysis of tramway tickets made in London. Needless to say, Mr. Gladstone's reply was in the negative. Alarming as the results may sound to the lay mind, they are really only what one would with certainty predict. We ought to know by this time that the microbes of disease abound on the surfaces of ourselves and everything we come in contact with, and that cultures taken from the surface of any common article of commerce, or from our own hands, when inoculated, would give the same disastrous results.

The analogies in structure between the trypanosoma of sleeping sickness and the treponema pallidum of syphilis have suggested the idea that atoxyl, which has been found so useful in the treatment of the former disease, might be equally specific for the latter. A large number of patients, over 150 in all, have been treated with the drug at the St. Louis Hospital, and Professor Hallopeau has communicated the results at the last meeting of the Académie de Médicine. He finds that atoxyl exercises a powerful action upon the specific virus of syphilis, and he is led to regard it as a third specific agent, equal, if not superior, to its predecessors. Secondary infections, such as condylomata and suppurations, appear to be refractory to the treatment. He is of the opinion that injections of the drug would probably cure the disease, if they could be given for a long enough period and in sufficient doses. Unfortunately symptoms of intolerance exhibit themselves after a varying number of injections. Such are gastro-intestinal pains, nausea, vomiting, pains in the limbs, and general *malaise*. Metchnikoff and Salmon have investigated the abortive power of atoxyl on the disease in monkeys. Seven monkeys were inoculated with the same syphilitic virus. Two of them were injected with atoxyl during the incubation period, and these did not develop the disease, whereas a chancre appeared in each of the remaining five monkeys.

It cannot be justly charged against the medical profession that it is in any way backward in clearly indicating to the legislators of the country such measures as it considers essential to the health and general wellbeing of the community. Since the present Government have come into power they have received more than one deputation on each of the many problems of hygiene which demand their consideration. Although these deputations have been cordially received, they have so far borne scanty fruit. Recently an influential deputation waited upon the President of the Board of Education to urge the immediate introduction of the teaching of hygiene and temperance into the curriculum of the Education Code. Lord Strathcona introduced the deputation, and among those who spoke were Sir Thomas Barlow, Mr. Pearce Gould, Professor Sims Woodhead, and Sir Victor Horsley. Mr. McKenna made a favourably reply to the many arguments urged, but begged a further delay in carrying out the objects for which the deputation pleaded, so that he might learn what the international conference to be held in London in August might have to tell him on the subject. He raised the same difficulty as his predecessor to a similar deputation, namely, that of getting teachers to teach the teachers, as well as teachers to teach the children. To equip the teachers with the necessary knowledge will, of course, require time, but there is no reason why it should take years, as Mr. McKenna suggested. If the people sufficiently realised the importance of these measures of hygiene to make them "election cries," ways and means would soon be found to carry them out, but so long as they are treated with apathy by the general public they are not likely to receive the urgent attention of the Government in power.

